# The Use of Dietary Supplements for Mental Health Among the Saudi Population

**DOI:** 10.1192/j.eurpsy.2022.1571

**Published:** 2022-09-01

**Authors:** D. Alateeq, G. Korayem, M. Alsubaie, F. Alsafi, S. Alsulaiman

**Affiliations:** 1 Princess Nourah Bint Abdulrahman University, College Of Medicine, Riyadh, Saudi Arabia; 2 King Abdullah bin Abdulaziz University Hospital, Department Of Internal Medicine, Riyadh, Saudi Arabia; 3 Princess Nourah Bint Abdulrahman University, Department Of Pharmacy Practice, College Of Pharmacy, Riyadh, Saudi Arabia; 4 King Abdullah bin Abdulaziz University Hospital, Pharmaceutical Care Services, Riyadh, Saudi Arabia; 5 Princess Nourah Bint Abdulrahman University, Health Sciences Research Center (hsrc), Riyadh, Saudi Arabia

**Keywords:** Dietary Supplements, mental health, sleep, Depression

## Abstract

**Introduction:**

Despite the limited evidence about the efficacy and safety of dietary supplement use for mental health, people tend to use them quite often. Generally the use of supplements among Saudi population shown to be prevalent, although limited studies that assessed their use for the improvement of mental health.

**Objectives:**

Identify the prevalence of dietary supplements use for mental health among the population in Saudi Arabia and also determine the factors that affect the use of dietary health supplements for mental health.

**Methods:**

A cross-sectional study of a convenience sample of 443 participants from various regions in Saudi Arabia. Questionnaire includes demographics, dietary use supplement assessment, and mental health assessment via the patient health questionnaire (PHQ-9), generalized anxiety disorder questionnaire (GAD-7), and insomnia severity index (ISI).

**Results:**

The prevalence of DS among the Saudi population reached 44%. Vitamin D and Melatonin were the most commonly reported DS used for mental health among the study population. The use of DS was associated with three times higher odds in patients who had previous mental health disorder diagnoses (OR 2.972; 95%CI; 1.602-5.515). The chance of using DS almost doubled in patients with subthreshold and moderate-severe insomnia (OR 1.930;95% CI 1.191-3.126) and (OR 2.485; 95% CI 1.247- 4.954) respectively.

**Conclusions:**

Despite the limited evidence about the efficacy and safety of dietary supplement use for mental health, people tend to use them quite often. Although the use of supplements among Saudi population shown to be prevalent, limited studies assessed their use for the improvement of mental health.

**Disclosure:**

No significant relationships.

